# A case of modern management of Morgagni‐Adam‐Stokes syndrome

**DOI:** 10.1002/ccr3.2384

**Published:** 2019-09-30

**Authors:** Maria Silvia Negroni, Francesca Furia, Francesca Bursi, Maria Paola Canevini, Stefano Carugo

**Affiliations:** ^1^ Division of Cardiology, Heart and Lung Department, San Paolo Hospital, ASST Santi Paolo and Carlo University of Milan Milan Italy; ^2^ Regional Centre of Epilepsy, Department of Health Science, San Paolo Hospital, ASST Santi Paolo and Carlo University of Milan Milan Italy

**Keywords:** cardiac resynchronization therapy with defibrillator, epilepsy, left bundle branch block, paroxysmal third‐degree AV block, syncope

## Abstract

Transient loss of consciousness initially diagnosed as epileptic seizures and then documented as paroxysmal atrioventricular block. Cardiac resynchronization and defibrillator therapy guided by a multimodality approach.

## Graphical abstract

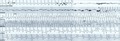

## 1 CASE PRESENTATION

We describe the case of a 67‐year‐old man who presented with episodes of transient loss of consciousness initially diagnosed as epileptic seizures and then documented as paroxysmal atrioventricular (AV) block.

The past medical history was unremarkable, except for systemic hypertension treated with Ramipril 5 mg daily and chronic stable left bundle branch block (LBBB). When the LBBB was discovered 6 years prior, a coronary angiogram was performed documenting normal coronary arteries and the echocardiogram was normal. The patient was referred to the epilepsy center of our hospital because of episodes of transient loss of consciousness that had occurred for 1 year. The episodes were previously diagnosed as seizures, but persisted despite antiepileptic therapy. The events were preceded by brief prodromes, particularly a sensation of warmth in the head and dizziness, and were characterized by stiffening and flexion of the upper limbs, dyspnea, and sialorrhea. They occurred when lying in the bed or turning over in bed. The frequency of episodes was initially monthly and then intensified weekly, and the duration was short (<1 minute). The patient also experienced episodes of hypotonic fall with rapid recovery. The interictal electroencephalogram (EEG) was normal. Apart from an EEG performed immediately after an episode which documented global slowing of the electrical activity, no episodes of transient loss of consciousness occurred during EEG recordings. Brain magnetic resonance (MR) imaging showed mild chronic vasculopathy. The patient was initially treated with Levetiracetam, with no effect. Lamotrigine and Clobazam were added but were ineffective. The persistence and increased frequency of the episodes despite a poly‐antiepileptic therapy and their unusual clinical presentation (ie, the correlation with the patient's position and the co‐occurrence of hypotonic falls with rapid recovery, which are not suggestive of seizures) led to hypothesize a cardiac cause, and the patient was sent for cardiology consultation to be evaluated for loop recorder implantation (ILR).

**Figure 1:**
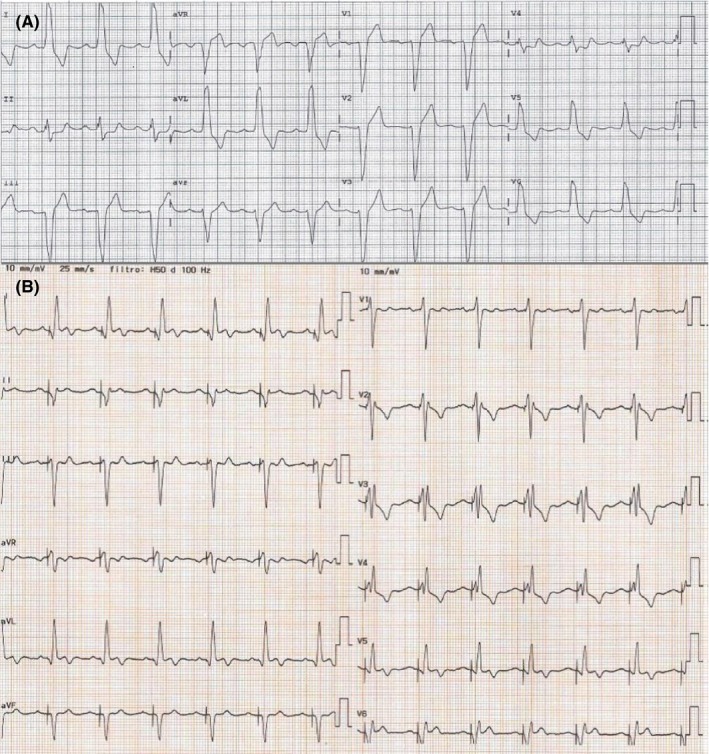
A, Baseline 12‐lead ECG recordings. Sinus rhythm, heart rate 77 bpm, PR 210 ms, LBBB with QRS duration 160 ms. B, Twelve‐lead ECG recordings on cardiac resynchronization therapy. Spontaneous sinus rhythm, heart rate 80 bpm, QRS morphology consistent with left ventricle only stimulation, and reduced duration of QRS at 120 ms

**Figure 2:**
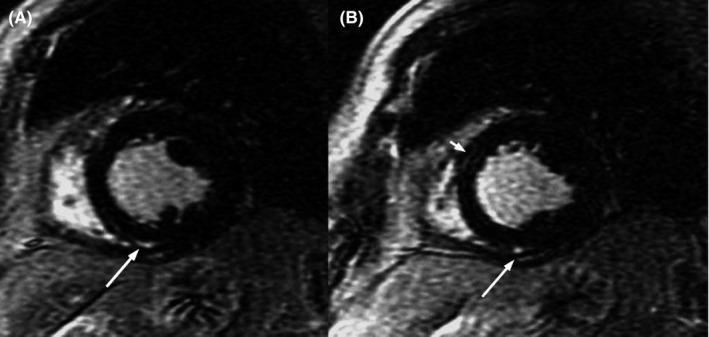
Short‐axis late gadolinium enhancement cardiac MR image showing intramyocardial late enhancement areas at the insertion of the posterior wall of the right ventricle on the interventricular septum (long arrow, panel A and B) and midmyocardial late enhancement area in the anterior interventricular septum at the base and middle level with non‐ischemic pattern indicating fibrosis (short arrow, panel B)

**Figure 3:**
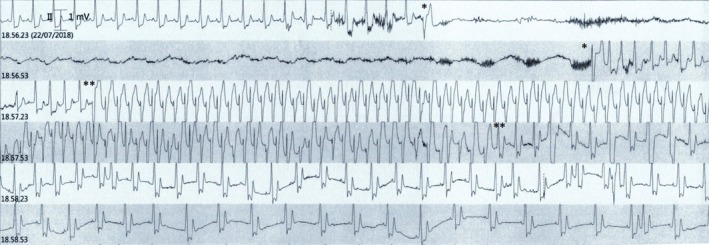
Telemetry monitoring tracings during the seizure episode shows an episode of paroxysmal AV block lasting 36 s initiated and terminated by a PVC (between single asterisks) and followed by a VT at 130 bpm lasting 40 s (between double asterisks)

The baseline 12‐lead electrocardiogram (ECG) confirmed sinus rhythm 65 bpm with stable LBBB (Figure 1A). The patient did not complain angina or palpitations. The transthoracic echocardiogram revealed a globally hypokinetic left ventricle with reduced left ventricular ejection fraction (LVEF 35%). The cardiac MR confirmed impaired LVEF (36%), associated with a marked desynchronization of contraction due to the conduction disturbance and a modest late gadolinium enhancement with an intramyocardial pattern (Figure 2). The MR did not show any signs of edema or acute myocarditis. The coronary angiogram was unchanged. We performed an electrophysiological study (EPS) with evidence of AH interval of 120 mseconds, HV interval of 60 mseconds, and antegrade Wenckebach point at 360 mseconds; no signs of sinus node dysfunction, carotid sinus massage negative bilaterally, no induction of supraventricular, or ventricular tachycardia. On telemetry monitoring during hospital stay, an episode of paroxysmal AV block with a ventricular asystole of 36 seconds was documented during an episode of seizure. The pause was introduced and closed by a premature ventricular contraction (PVC), and after some sinus beats conducted with LBBB, an episode of ventricular tachycardia (VT) at 130 bpm was observed, with spontaneous resolution after 40 seconds (Figure 3). The patient underwent implantation of a three transvenous leads device, respectively in the right atrium, in the right ventricle for defibrillation and antitachycardia pacing and in a posterolateral branch of the coronary sinus (cardiac resynchronization therapy with defibrillator or CRT‐D). At 1‐month postimplantation visit, there was constant left ventricular stimulation according to the adaptive CRT algorithm of the device Medtronic Claria Quad (Figure 1B), and the echocardiogram revealed recovery to a normal LVEF. The patient continued therapy with ACE‐ inhibitor and beta‐blocker with a gradual weaning from antiepileptic therapy. At 12 months, no paroxysmal AV block, neither VT were recorded by the device monitor and no recurrent episode of seizure occurred.

## 2 DISCUSSION

Transient loss of consciousness is a common chief complaint of patients presenting to an Emergency Department. It comprises a heterogeneous group of disorders, including epileptic seizures and various types of syncope.^1^ Reflex syncope and focal seizures with secondary generalization share similar manifestations. It is therefore often difficult to distinguish epileptic seizures from convulsive syncope based solely on the clinical history. The diagnosis of epilepsy is often based only on the clinical picture, as the interictal EEG can be normal.^2^ Convulsive syncope is a common cause of misdiagnosis in patients with a transient loss of consciousness. This misdiagnosis contributes significantly to the number of patients with a questionable diagnosis of epilepsy and to those with apparently drug‐resistant epilepsy. The use of ILR to document heart rate changes during events has proven to be useful,^3^ and their employment in patients with drug‐resistant epilepsy has been added in the latest version of syncope guidelines (class IIb indication).^4^

Paroxysmal third‐degree AV block is defined as the sudden and unexpected repetitive block of the atrial impulse on its way to the ventricle with consequent asystolic ventricular pause due to delayed emergence of a satisfactory escape rhythm. It is a known cause of syncope and potentially sudden cardiac death (SCD) which may be prevented, if promptly diagnosed, with permanent pacemaker implantation (PPM). Three types of paroxysmal AV block have been described: extrinsic vagal paroxysmal AV block, extrinsic idiopathic paroxysmal AV block, and intrinsic paroxysmal AV block with distinct clinical and electrophysiological features.^5^ Extrinsic vagal paroxysmal AV block is linked to the effect of the parasympathetic nervous system on cardiac conduction and is one of the mechanisms involved in “reflex syncope”. Extrinsic idiopathic paroxysmal AV block is associated with low levels of endogenous adenosine and is supposed to be one of the mechanisms involved in “low‐adenosine syncope”. Intrinsic paroxysmal AV block due to an intrinsic disease of the AV conduction system (Stokes‐Adams attack) usually occurs in patients older than 60 years with an underlying structural heart disease. It is associated with abnormal ECG, namely bundle branch block. This block is often initiated and ends with an extrasystole, and the frequency of the sinus node stimulation may be increased (tachy‐dependent AV block) or decreased (brady‐dependent AV block). The onset of syncopal episodes typically occurs within 1 year before the ECG diagnosis. Most of these characteristics were present in our patient suggesting intrinsic AV block. Furthermore, the loss of consciousness episodes occurred while the patient was lying in supine position, which is a high‐risk feature as well as if its occurrence is during exertion. Electrocardiographically, the sudden onset of AV block explains why prodomes are absent or shorter than 5 seconds as in the present case.

It is well known that patients with syncope and bundle branch block are at increased risk for developing advanced AV block, such that current European Society of Cardiology guidelines recommend that the EPS should be performed in this patient population (class IIa).^4^ Furthermore, the EPS allows to exclude other mechanisms of syncope, for example, tachyarrhythmias. The EPS is considered diagnostic for intrinsic AV block when the baseline HV interval is ≥70 mseconds or second‐ or third‐degree His‐Purkinje block appears during incremental atrial pacing or after pharmacologic challenge with intravenous class I antiarrhythmics. However, despite an acceptable positive predictive value, the sensitivity of EPS is low and a negative EPS may not rule out the presence of paroxysmal AV block, as happened in our patient. Therefore, in patients with bundle branch block prolonged monitoring with ILR may be helpful to establish the diagnosis. LBBB, seldom isolated, is mostly associated with structural heart disease and has a prevalence of 1% in the general population. There is evidence that LBBB is not only a bystander or a mere marker of the severity of left heart disease, but may have a role in the genesis of dilated idiopathic nonischemic cardiomyopathy through several mechanisms including dyssynchrony, unequal distribution of myocardial workload during the cardiac cycle and altered blood flow and metabolism.^6^ When LBBB was first discovered in our patient, systolic function was normal by echocardiography. However, 6 years of prolonged exposure to LBBB likely favored adverse remodeling and possibly myocyte loss and replacement by fibrosis. LBBB‐induced cardiomyopathy responds dramatically to CRT^7^; however, in some subjects the risk of ventricular tachycardia, heart failure hospitalization, and death remains despite improvement of systolic function.^8^

Assessment of midwall fibrosis with late gadolinium enhancement cardiac MR provides independent prognostic information beyond LVEF in patients with cardiomyopathy.^9^ Fibrosis is associated with contractile impairment and is a largely recognized substrate for ventricular reentrant arrhythmia. Therefore, cardiac MR imaging may play a role in estimating CRT response and SCD risk before implant and may help to identify those individuals who would best benefit of CRT defibrillators rather than CRT pacemakers only, albeit this has not been encompassed in the current guidelines yet. We do not know the exact mechanism of myocardial fibrosis in this patient: We may hypothesize a role of the LBBB, a previous unrecognized myocarditis, or an effect of the ischemic insults due to the repetitive asystolic periods, but the pattern of distribution of scar by MR was nonischemic. Nevertheless, once the final diagnosis of intrinsic AV block is made a bicameral PPM is mandatory. Because of concomitant decreased LV systolic function associated with LBBB, we elected to implant CRT with successful recovery of LVEF at 1 month. The choice of implanting a device encompassing defibrillator function was not merely driven by the VT occurring during the index event, yet on the presence of low LVEF and finding of myocardial scar at the cardiac MR. Indeed, the post‐AV block arrhythmia was likely secondary to an ischemic insult due to prolonged asystole. The device was programed with adaptive algorithm which periodically verifies the intrinsic conduction and dynamically corrects the pacing, providing left only pacing if AV conduction is preserved or biventricular pacing if necessary.^10^

Our patient was a CRT responder as shown by the significant postimplantation narrowing in QRS duration, probably the main determinant of echocardiographic reverse remodeling as shown by the early improvement in LVEF. Electrical and mechanical resynchronization is known to be associated with a favorable outcome.^10^, ^11^, ^12^

## CONFLICT OF INTEREST

The authors have no conflicts of interest to disclose.

## AUTHOR CONTRIBUTIONS

MSN: treated the patient, was responsible for collecting and interpretation of data and drafting of manuscript, had full access to all the data in the study and takes responsibility for the integrity and the accuracy of the data. FB: was responsible for doing echocardiography and helped in drafting and critical revision of the manuscript. FF and MPC: were responsible for neurologic consultations and critical revision of the manuscript. SC: was responsible for critical revision of the manuscript for content and supervision. All authors reviewed and contributed to the final version of this case report. All authors read and approved the final manuscript.
